# High EGFR and low p-Akt expression is associated with better outcome after nimotuzumab-containing treatment in esophageal cancer patients: preliminary clinical result and testable hypothesis

**DOI:** 10.18632/oncotarget.4367

**Published:** 2015-06-08

**Authors:** Chun-yu Wang, Jia-ying Deng, Xu-wei Cai, Xiao-long Fu, Yuan Li, Xiao-yan Zhou, Xiang-hua Wu, Xi-chun Hu, Min Fan, Jia-qing Xiang, Ya-wei Zhang, Hai-quan Chen, Rolando Perez, Guo-liang Jiang, Kuai-le Zhao

**Affiliations:** ^1^ Department of Radiation Oncology, Fudan University Shanghai Cancer Center, Department of Oncology, Shanghai Medical College, Fudan University, Shanghai, China; ^2^ Department of Radiation Oncology, Shanghai Chest Hospital, Shanghai Jiao Tong University, Shanghai, China; ^3^ Department of Pathology, Fudan University Shanghai Cancer Center, Shanghai, China; ^4^ Department of Medical Oncology, Fudan University Shanghai Cancer Center, Shanghai, China; ^5^ Department of Thoracic Surgery, Fudan University Shanghai Cancer Center, Shanghai, China; ^6^ Center of Molecular Immunology, Havana, Cuba

**Keywords:** esophageal squamous cell carcinoma, EGFR, p-Akt, p-Erk, monoclonal antibody

## Abstract

The epidermal growth factor receptor (EGFR) is widely overexpressed in esophageal squamous cell carcinoma (ESCC) and it results is associated with a poor prognosis. Identifying the subgroup of ESCC patients who are sensitive to EGFR-targeted therapy is a key point to facilitate its medical use.

We retrospectively analyzed 32 ESCC patients treated with the combination of nimotuzumab (h-R3) and radiotherapy (RT) or chemoradiotherapy (CRT). Expression of EGFR and phosphorylated proteins associated with EGFR signaling pathway, i.e. p-Akt and p-Erk, were assessed with immunohistochemistry (IHC) for all patients. Correlations between these proteins' expression levels and overall survival (OS) were assessed.

High expression of EGFR, p-Akt and p-Erk was detected in 53.1% (17/32), 54.8% (17/31) and 59.4% (19/32) of tumors respectively. No significant differences in OS were found between high EGFR, p-Akt and p-Erk expression groups and their respective counterparts. Of note, significantly better overall survival was observed in patients with coexistence of high EGFR expression and low p-Akt expression (*p* = 0.030).

Our data allowed us to put forward a hypothesis that high EGFR and low p-Akt expression may predict a clinical benefit of EGFR antagonists such as nimotuzumab combined with RT or CRT. This can be discussed in the terms of oncogene addiction and synthetic lethality concepts. This hypothesis can be further tested in larger groups of patients.

## INTRODUCTION

In 2012, an estimated 455,800 new esophageal cancer cases and 400,200 deaths occurred worldwide. The highest rates are found in Eastern Asia and in Eastern and Southern Africa and the lowest rates are found in Western Africa [1]. Although improved surgical techniques and combined modalities (surgery, chemotherapy, and radiotherapy) have been developed and introduced into clinical practice over the last decade, the overall survival (OS) for esophageal squamous cell carcinoma (ESCC) remains unsatisfying, with reported five-year OS rates of only 20% to 47% [2-4]. In order to improve this situation far from ideal, targeted therapy has been widely investigated in ESCC.

It has been demonstrated that overexpression of epidermal growth factor receptor (EGFR), which can be detected in 50%-70% of esophageal cancer cases, is correlated with poor prognosis [5-8]. With a ligand binding to its extracellular domain, EGFR can be activated and subsequently promotes proliferation, survival, angiogenesis, and resistance to radiotherapy and chemotherapy mainly through two signaling pathways: the RAS/RAF/MEK/Erk/MAPK and the PI3K/Akt/mTOR axes [7, 9-11].

It is certainly not unreasonable to assume that EGFR blockage might be an effective therapy for ESCC, but no improvement in clinical efficacy was found in unselected esophageal cancer patients according to the results of the randomized phase II/III SCOPE1 study in which adding cetuximab (an anti-EGFR antagonist monoclonal antibody) to standard definitive chemoradiotherapy was evaluated [12]. Similarly, the clinical trial COG found no improvement on overall survival for gefitinib as a second-line treatment in esophageal cancer [13], however palliative benefits were reported in these difficult-to-treat patients with short life expectancy [13]. Therefore, identifying the subgroup of ESCC patients who are sensitive to EGFR-targeted therapy represents the key point to increase its efficacy.

Nimotuzumab (h-R3) is an anti-EGFR antagonist humanized monoclonal antibody showing promising performance in head and neck cancer and esophageal cancer with excellent safety profile and limited side effect [14, 15]. In a previously published retrospective study, we found that combining h-R3 with radiotherapy (RT) or chemoradiotherapy (CRT) resulted in moderate benefit on OS in patients with advanced ESCC [16]. Subsequently, the question whether the expression level of EGFR, phosphorylated (p-) Akt or phosphorylated (p-) Erk could be used as factors to predict the overall survival (OS) in these patients was investigated. We hypothesized that patients with high EGFR expression would have a better overall survival with the EGFR inhibitor h-R3 antibody, compared with the patients with low expression.

## RESULTS

### Clinical characteristics and outcome

Pre-therapeutic tumor materials were available from 32 patients whose clinical characteristics were shown in Table 1. Among these patients, 30 (93.8%) of them were treatment-naïve while the other 2 (6.3%) had relapsed disease after surgery or radiation. There were 25 patients (78.1%) who received concurrent h-R3 and CRT and 7 patients (21.9%) received concurrent h-R3 and RT. Dose of 100mg, 200mg and 400mg of h-R3 were given to 5, 22 and 5 patients per week respectively. The median duration of h-R3 treatment was 6 weeks (interval of 3 to 9 weeks). 8 out of 32 patients received radiation equal to or less than 60Gy, while 24 patients received more than 60Gy. All patients received 3-dimensional conformal radiation therapy (3D-CRT) or intensity-modulated radiation therapy (IMRT) with a 6-MV X-ray beam. The median OS was 35.1 months (95% CI, 22.1 months to 48.0 months) with a median follow up time of 40.8 months.

**Table 1:** Clinical characteristics

Variable	Number of patients (%) *N*=32
Age (years)	
≤ 65	21(65.6)
> 65	11(34.4)
Gender	
Male	26(81.3)
Female	6(18.8)
Tumor stage^a^	
Stage I-II	9(28.1)
Stage III-IV+ relapse	23(71.9)
Location of tumor	
Cervical + upper thoracic	12(37.5)
Middle +lower thoracic	16(50.0)
Double primary esophageal cancer	4(12.5)
Tumor length (for treatment-naive)	
≤5cm	15(50%)
>5cm	15(50%)
Chemotherapy	
Irradiation alone+h-R3	7(21.9)
Chemoradiotherapy+h-R3	25(78.1)
Radiation dose	
≤60Gy	8(25)
>60Gy	24(75)

a UICC 7^th^ edition of esophagus and esophagogastric junction cancer.

### Protein expression

**Figure 1:**
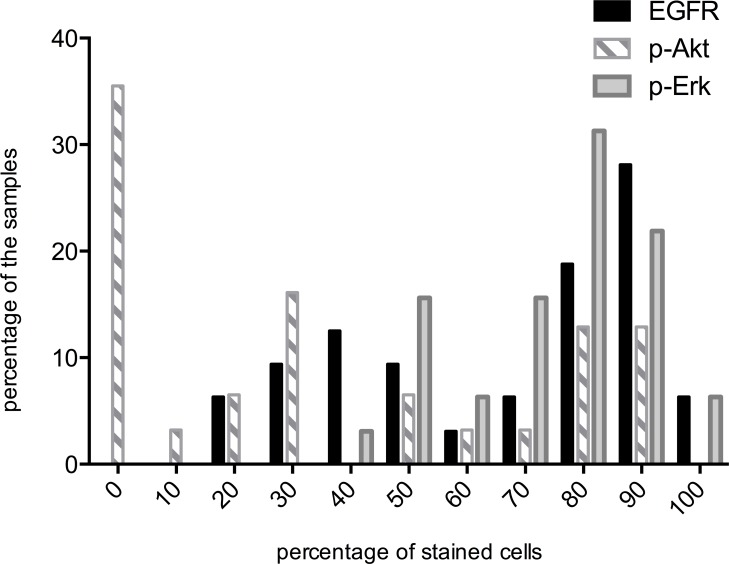
Distribution of EGFR, p-Akt and p-Erk positive cells across all tumor samples Percentage of samples (Y-axis). Percentages of stained cells (X-axis). The median percentages were 80, 30 and 80 for EGFR, p-Akt and p-Erk, respectively. High : IHC score ≥ median score; Low: IHC score <median score.

The percentages of stained cells (IHC scores) for EGFR, p-Akt and p-Erk are shown in Figure 1. Median values were 80%, 30% and 80% respectively. Studied proteins showed different expression patterns in the evaluated tumor samples. Among the 32 samples examined for EGFR expression, 15 (46.9%) were classified as EGFR low and 17 (53.1%) exhibited high EGFR expression. 4 out of 17 patients belonging to the EGFR-high subgroup underwent radiation combined with h-R3 and the others were treated with the combination of chemoradiotherapy plus h-R3. No correlation was found between EGFR expression and age, gender, tumor length, or tumor stage.

Assessment for p-Akt expression was performed in 31 tumor samples, 14 (45.2%) of them were classified as low expression while 17 (54.8%) exhibited high expression. There was no correlation between the expression of EGFR and p-Akt (*p* = 0.224), 8 patients were EGFR low/p-Akt low, 11 patients were EGFR high/p-Akt high, 6 patients were EGFR high/p-Akt low and 6 patients were EGFR low/p-Akt high. Furthermore, there was not significant correlation between p-Akt expression and age, gender, tumor length and tumor stage.

For p-Erk, 13 (40.6%) tumor samples exhibited low expression and 19 (59.4%) samples showed high expression. There were 8 patients EGFR low/p-Erk low, 12 patients EGFR high/p-Erk high, 5 patients EGFR high/p-Erk low and 7 EGFR low/p-Erk high. Statistical analysis did not show any significant correlation between p-Erk and age, gender, tumor length, tumor stage and EGFR.

### Correlation between protein expression and OS

**Figure 2:**
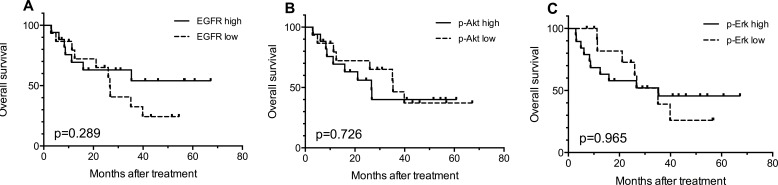
Kaplan-Meier curves for overall survival (OS) according to the expression levels of EGFR (A), p-Akt (B) and p-Erk (C) in 32 available tumor biopsy samples EGFR high: IHC score ≥80 (median score); p-Akt high: IHC score≥ 30 (median score), p-Erk high: IHC score ≥80 (median score). IHC score: the percentages of stained positive cells.

Kaplan-Meier survival curves were used to estimate the OS. As shown in Figure 2A, there was a trend (*p* = 0.289) that patients expressing high EGFR had better OS compared with patients with low expression. In addition, no significant correlation between the protein expressions and OS for p-Akt (*p* = 0.897, Figure 2B) and p-Erk (*p* = 0.965, Figure 2C) was found. Results of multivariate analysis (Cox regression) aiming to determine the independent prognostic values of different variables, including age, gender, with chemotherapy or not, tumor stage, radiation dose, EGFR expression, p-Erk expression and p-Akt expression, did not show statistically significant findings.

**Figure 3:**
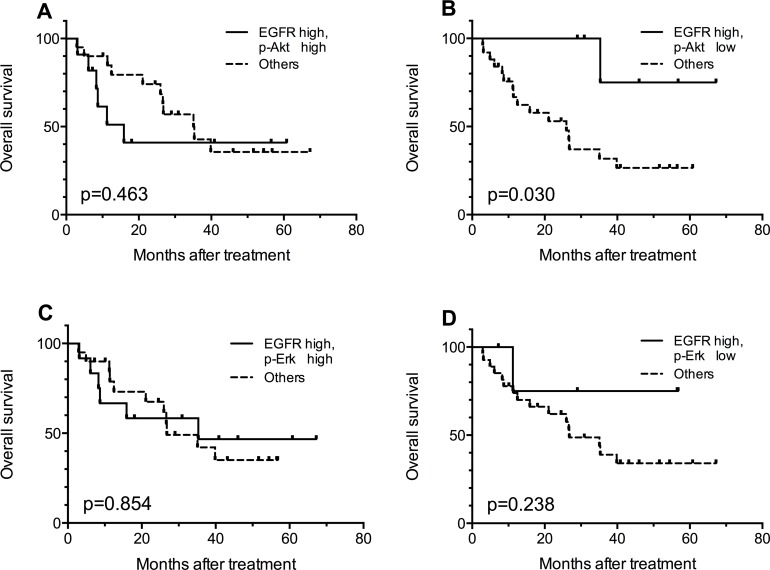
Kaplan-Meier curves for comparisons between EGFR high patients with certain p-Akt or p-Erk expression status and the rest of the patients Patients with EGFR high/ p-Akt low tumors got better overall survival **(B)**, and other comparisons showed no statistically significant results **(A, C, D)**.

In order to identify a patient subgroup that is most likely to benefit from the treatment, combined effect of biomarkers were evaluated. Given the possible trend that patients with high EGFR expression might benefit more from h-R3 treatment (Figure 2A), we further compared the OS of patients with high EGFR expression and certain p-Akt or p-Erk expression status with that of the rest of the patients (e.g. patients with high-EGFR and high p-Akt versus the entirety of other patients). As shown in Figure 3A and 3B, the patients with high EGFR and low p-Akt had significantly better survival (*p* = 0.030) compared with other sub-groups; this was not the case for patients EGFR high/p-Akt high (*p* = 0.463). On the other hand, analyses considering high EGFR expression and p-Erk status did not show any statistically significant findings (Figure 3C, 3D).

## DISCUSSION

In our study focusing on ESCC patients, we measured the expression of EGFR and two phosphorylated proteins respectively essential for the activation of its two main downstream signaling pathways, ie p-Akt and p-Erk, in order to assess their potentiality to predict the outcome after treatment with nimotuzumab in these patients. Our results suggest that coexistence of high EGFR expression and low p-Akt expression in individuals may be associated with better OS after being treated with h-R3 combined with RT or CRT.

Overexpression of EGFR has been reported to be associated with poor prognosis in experimental and clinical settings [6, 10, 17]. Accordingly, several EGFR inhibitors have been developed during the last ten years, including monoclonal antibodies such as cetuximab and panitumumab, and small tyrosine kinase inhibitors such as gefitinib and erlotinib. However, results less favorable than expectations were observed in clinical trials investigating the combination of cetuximab or gefitinib and concurrent conventional chemotherapy in esophageal cancer [12, 13]. Although no improvement in OS was found for the entire population investigated, results of these trials suggest that patients with certain clinical and/or biological characteristics may do better with EGFR inhibitors and these characteristics especially certain molecular biomarkers may be useful for stratification and outcome prediction.

Given that predictive markers for other epithelial cancers such as mutations in KRAS, EGFR or BRAF are rarely detected in esophageal cancers, EGFR expression has been predominantly investigated for its potentiality of predicting treatment outcome for esophageal cancers [18-21]. However, Liang J et al demonstrated that EGFR failed to predict the outcome in a phase II study combining h-R3 and radiation therapy in ESCC [22]. Furthermore, no correlation between EGFR expression and outcome was observed in patients with gastroesophageal (GE) junction and gastric adenocarcinoma treated with erlotinib or cetuximab [23, 24].

As to p-Akt and p-Erk alone, a few clinical researches had tested their contribution in regimens containing EGFR inhibitors. In a Phase II trial of erlotinib in gastroesophageal junction and gastric adenocarcinomas, no correlation was found between p-Akt and clinical outcome [24]. In experimental models, Fichter et al reported in OE21 cells (ESCC cell line overexpressing EGFR), a significant reduction of cell viability up to about 50% occurred upon treatment with erlotinib, gefitinib or lapatinib. All three inhibitors reduced p-Akt level but p-Erk expression remained the same as pretreatment [25]. Mimura et al also demonstrated p-Akt expression was enhanced to a large extent by EGF, whereas p-Erk expression was marginally increased in response to EGF when the EGFR-overexpressing ESCC cell line KYSE30 was treated with EGF [26]. Hitherto, we may deduce that the EGFR/p-Akt pathway is more crucial than the EGFR/p-Erk pathway in ESCC cells.

Akt is a very common effector for numerous upstream signals such as VEGF, and other HER family members such as HER2 and HER3 [10, 25, 27, 28]. In ESCC, certain membrane receptors other than the EGFR may also be able to activate the Akt. Base line p-Akt level has been demonstrated to have an inverse correlation with patients' survival [29]. Yoshioka A et al reported that low expression of p-Akt was associated with a better prognosis in the ESCC patients who received chemotherapy combined with surgery [30].

**Figure 4:**
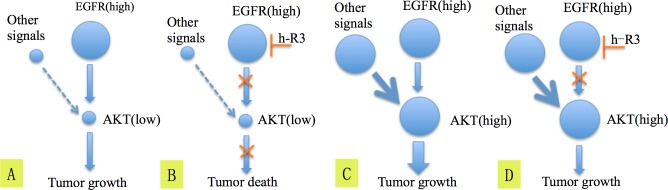
The hypothesis explaining the mechanism of blockage of EGFR by h-R3 and inactivation of Akt which lead to better survival in patients having tumors EGFR high/p-Akt low **(A, B), C. and D.** show the possible mechanism explaining why EGFR high/p-Akt high patients didn't get benefit from the h-R3 treatment.

Hitherto, we bring out a hypothesis about the mechanism how h-R3 works on EGFR high/p-Akt low patients (Figure 4). For tumors EGFR high/p-Akt low, receptor tyrosine kinases (RTKs) besides EGFR have limited influence on activation of Akt (Figure 4A). Subsequently, the blockage of EGFR by h-R3 reduces the activation of Akt and result in tumor death (Figure 4B). In contrast, if the tumor is EGFR high/p-Akt high, the RTK signals besides EGFR may play important roles in activating Akt (Figure 4C). Consequently, the Akt protein can still be activated by other signals despite the EGFR blockage by h-R3, and this may lead to tumor progression (Figure 4D). All above can be explained in the terms of oncogene addiction and synthetic lethality concepts, which have been brought out in other cancers [31-34]. For EGFR addiction, a fraction of primary ESCC cells apparently are dependent on EGFR activity for survival. The cells die when suddenly deprived of EGFR. On the other hand, the weakness of RTKs and inhibition of EGFR resembles synthetic lethality.

For EGFR high/p-Akt high patients, the activation of PI3K-Akt-mTOR due to HER2 or HER3 overexpression has been reported to be one of the possible mechanisms causing resistance to EGFR blockage [35]. These results are in agreement with the hypothesis postulated in this paper. We can not exclude so far the possibility that tumors EGFR high/p-Akt high would be sensitive to the combination treatment using anti-EGFR inhibitors plus either anti-HER2, anti-HER3 or anti-mTOR antagonists [36].

It should be noted that this study had examined only a group of patients, which were treated with h-R3 and RT or CRT. We didn't refer to the patients treated with hR-3 only or RT/CRT only. However, we are currently conducting a prospective, randomized and double-blinded clinical trial (NCT01232374) to compare concurrent chemoradiation and h-R3 to chemoradiation alone in esophageal SCC that may distinguish effects from RT/CRT from those of h-R3. Furthermore, our study just included a small series of patients with a retrospective study. So we just report the preliminary clinical result and bring out the testable hypothesis.

In conclusion, in the present study was found a possible relationship between the expression levels of EGFR, p-Akt, and p-Erk and the outcome after the combination treatment of h-R3 plus CRT or RT for a group of 32 ESCC patients. Patients with EGFR high/p-Akt low tumors had better survival. The possibility of using two potential protein biomarkers along for outcome prediction might be a direct consequence of the complicated phenomena associated with growth factor signaling networks [37]. More clinical data are needed to confirm this result and to determine the subgroup of ESCC patients who would be possibly more sensitive to the treatment with nimotuzumab or other anti-EGFR drugs.

## MATERIALS AND METHODS

### Patient selection

From December 2008 to September 2011, we retrospectively analyzed 66 patients treated with h-R3 concurrently with RT or CRT, and corresponding safety and 2-year OS data were published previously [16]. The 32 patients selected in the current study were a subset of patients whose tumor samples (formalin-fixed and paraffin-embedded) had been taken before the treatment. As reported earlier, eligibility criteria for this study also included the following: histological evidence of invasive squamous-cell carcinoma of the esophagus, informed consent to receive irradiation (RT) or chemoradiation (CRT), at least one dose of h-R3 without prior treatment with other targeted agents, white blood cell count of at least 4.0×10^9^/L, and platelet count of at least 10.0 ×10^9^/L.

### Immunohistochemistry (IHC) assay

IHC assay was performed using conventional methods. Briefly, after paraffin microtome sectioning (4 micrometer) and deparaffinization, endogenous peroxidase was blocked by 3% H_2_O_2_, and antigen retrieval was completed after being heated in citrate buffer using microwave oven. The sections were incubated with primary antibody over-night, then with secondary antibody. Finally the sections were processed with diaminobenzidine (DAB) and counterstained with hematoxylin. Antibodies against EGFR (Cell Signaling, USA; working concentration: 1:100), p-Akt (Cell Signaling, USA; working concentration: 1:40) and p-Erk (Abcam Ltd, USA; working concentration: 1:50) were used.

### Reactivity score and interpretation of the immunohistochemical staining

For a semi-quantitative analysis of protein expression, all stained slides were assessed by two referenced pathologists (Li Y and Zhou XY, both are Professors of pathology from and verified by Fudan University Shanghai Cancer Center) who were blind to the survival data. Tumor cells were scored by the percentage of the stained cells from 0 to 100 in increments of 10. For positive staining in the nuclei, the most intensively stained region would be first selected with low-power magnification. The percentage of stained cells would then be calculated by counting 10 consecutive high-power fields (×400). For positive staining on the cell membrane or in the plasma, the percentage of cells stained was obtained by microscopically examining/analyzing the entire tissue at low magnification (×100). To evaluate the relationship between protein expression levels and OS, patients were classified into high or low expression group based on whether the percentage of positive cells (IHC staining) was higher or lower than the median value obtained for each target protein, respectively[38].

### Statistical analysis

The Kaplan-Meier model was used to estimate the OS. Differences in survival between subgroups were compared by log-rank test. Patient groups were compared with the Pearson's χ^2^ test or Fisher's exact test when appropriate. All statistical analyses were performed using SPSS software (version 19.0, SPSS Inc. Chicago, IL). A two-sided *p* value < 0.05 was considered to be significant.
